# What Is an Accident?

**Published:** 1913-06

**Authors:** Charles Corfield

**Affiliations:** the Middle Temple


### WHAT IS AN ACCIDENT?

BY

CHARLES CORFIELD, of the Middle Temple, Barrister-at-Law, M.R.C.S., L.R.C.P.

---

THE consideration of accidents is a matter which comes to most of us sometime or other in our professional work, mainly, of course, arising out of the Workmen's Compensation Act, but sometimes under common law as well; and therefore a review of those decisions which have helped to define what an accident is may not be out of place at a medical meeting.

It is curious that neither of the Workmen's Compensation Acts contained this definition, so that the meaning of the term has tended to vary case by case. Prior to these Acts little difficulty had ever arisen in common law cases of negligence, as running-down accidents or railway accidents, and as regards workmen before these Acts negligence was the only claim for accident that could be sustained by a servant against a master. But these statutory enactments inaugurated a new principle in English law, and so gave a legal subtlety to the word "accident" that it never had before. The first Act was limited in scope, but the 1906 Act was of general application, and also made certain diseases statutory accidents as well. The first definition of an accident that I am able to find is in Stephen's *Commentaries*. He says in effect an accident is any mishap or untoward event which is not expected or designed, whether it be caused by the negligence of another person or not. This definition falls very short of the modern interpretation of accident. Many occurrences which cause injury are not unexpected, but a highly probable act of the workman himself. As the late Lord MacNaughten once said, a man may injure himself by doing some stupid thing and it is called an accident. The definition of Sir James Stephen, then, as it excludes the consequences of carelessness from the meaning of the word accident, fails at the very outset as a guide to the interpretation of the term.

Another definition might be taken from a class of case where previous to the Workmen's Compensation Act the word accident was sometimes likely to come under review, namely in insurance and marine cases. Thus we find Mr. Justice Willes in 1868 holding that an exception in a charter party against riots and strikes or *any other accident* does not include a snowstorm. He says an accident is not an ordinary occurrence, but something which happens out of the ordinary course of things. A snowstorm, however, is one of the ordinary operations of Nature, and may be described rather as an *incident* than an accident.

Yet in the comparatively early days of the Workmen's Compensation Act we find that the effects of frost were held to constitute an accident, and later on the death of a workman by lightning was similarly held to be an accident too. So as judgment succeeded judgment the meaning and scope of the word became amplified and extended.

Again, in the well-known case of Fenton *v.* Thorley, it was laid down that to be an accident within the meaning of the Act there must be something fortuitous or unexpected in the occurrence. Fenton was a man in the employ of Messrs. Thorley. Whilst at work one day upon a machine he felt something which in giving evidence he described as a tear in his inside, and it was found that he had a rupture. In the medical evidence it was said that the hernia was a physiological one, which I suppose meant a congenital condition. In other words, that it had existed before, but that a severe strain had brought it down. Here we have an extension of the word accident to a condition which existed as a concealed defect, and which another workman without this congenital weakness doing the same work would not have contracted ; and this is the first important departure from the word accident in its restricted sense.

The next case of importance which enlarged the scope of this much-debated word was Brinton *v.* Turvey. A wool sorter contracted anthrax. (This, I may add, was before it was scheduled as an industrial disease.) In the course of the case on appeal, Lord Robertson said: "Anthrax is a disease, and unless the contraction of an infectious disease is an accident in the sense of the Act, I do not see how this judgment can stand. If it does stand, then in every case in which a man dies of any infectious disease contracted in the course of his employment all he has got to do is to get a doctor to say that a micro-organism did it, and the accident is there." The majority of the "House" was against Lord Robertson; so an infectious disease was by a legal decision from the highest tribunal included in the category of possible accidents under the Workmen's Compensation Act.

We now reach a further process of development in the meaning of the much-suffering word, for we come to a class of case where disease has been produced by a continual process rather than by a definite event, and yet the result has been held to be an accident.

Thus in Ismay & Co. *v.* Williamson a workman was employed as a trimmer on board the s.s. *Majestic*. He was a man of feeble physique and health. His work lay in the stokehold, where the heat was great. He developed on the second day symptoms of heat stroke and dropped in a faint. He was carried to the ship's hospital, and became violent and died from exhaustion two hours after leaving the stokehold. The condition of the stokehold was normal and ventilation good, and other stokers had worked for years in such conditions without any serious effects.

By a majority the House of Lords decided that this was death arising from accident. In the course of his judgment, the Lord Chancellor said what killed the man was a heat stroke coming suddenly and unexpectedly upon him while he was at work. Such a stroke is an unusual effect of a known cause, often no doubt threatened, but generally averted by precautions which experience in this instance had not taught. It was an unlooked-for mishap in the course of his employment. The judgment of Lord MacNaughten on this occasion is interesting, though he was in the minority. He said: "It does not seem to me to come under the description of a personal injury by accident. The death was due to the physical state of the workman more than the nature of his employment."

Now, side by side with these two cases I have mentioned there are two other cases also of considerable interest, viz. Brodrick *v.* London County Council and Eke *v.* Hart Dyke (1910). In the first case the workman, according to the medical evidence, contracted enteritis from inhaling sewer gas for a period of six hours, and the result of the enteritis was to accelerate existing heart disease, and so precipitate the incapacity from heart trouble which sooner or later would have overtaken him. This was held by the Court of Appeal not to be a case of accident.

The case of Eke *v.* Hart Dyke, which I think is one of the last leading cases on this subject, still further confirms this decision I have just mentioned. It was a case in which the caretaker of an empty house was told to lay open the drains, manholes and cesspools for inspection. The man did this on several occasions, and becoming ill, died in the following October from poisoning contracted from the drains. It was held that this was not an accident. The Master of the Rolls, in giving judgment, referred to the anthrax case, and pointed out that in that instance the anthrax was due to the circumstance that at a particular time and at a particular place the infection came into the man's eye, and his lordship appeared to consider that that constituted a satisfactory ground for distinction. But if a particular event happens at a particular time and at a particular place, what about Ismay's, the stoker's case, where the man had worked two turns already that day in the stokehold and part of a third turn. Was there in that case any particular time at which the occurrence of a given event could be definitely ascertained and distinguished as a separate occurrence ? His collapse no doubt took place at a definite time, but his collapse was the result of an accident, and not the accident itself, because it was, so to speak, presumably the cumulative effect of several hours' excessive heat. If the test of a definite event at a definite time has to be applied, what length of time is sufficient for the purpose ? Must it be an instantaneous effect, or may it be a slowly-operating process covering a period of time ? If so, how long a period ? In Ismay's case the man had been working for several hours on the second day, and there is nothing such as ventilation block which fixes any definite event during these periods. One naturally asks why is the six hours during which Brodrick worked in the sewer too indefinite, while the longer period during which Ismay's worked is definite enough.

These cases I have above illustrated are sufficient to show how difficult the question of accident is. The Master of the Rolls himself says the words injury by accident have undoubtedly created great difficulty. "I feel," said a recent Lord Chancellor, "that in construing this Act of Parliament there is a risk of frustrating it by excess of subtlety, which I am anxious to avoid." These difficulties are not due to a conventional inflexibility of the judicial mind, but to a trait inherent to any words that define by limitation, namely that wherever the boundary-line be drawn some cases will always be found to fall extremely near on either side. The solution of this vexed question might be attempted in several ways. Some day an ingenious definition of the word accident might be evolved which will steer between these dangers I have outlined above ; then more importance might be attached, not to the quality of the blow, so to speak, but to the condition of the recipient. Something, in other words, of the principle of contributory negligence might be introduced, and it could be made a good defence to a claim to show that the workman had a concealed defect which contributed equally to the condition, such as an aneurysm, a congenital hernia, and so on.

These restrictions would, however, if pushed very far, tend, I am afraid, to defeat the purpose of the Acts themselves, and therefore would not be tolerated.

Another suggestion is that all employees should undergo a physical examination, which is already done in the case of railway and other big companies, and as they pay a fee, from the point of view of the doctor such a plan has much to recommend it. This, however, without the privilege of contracting out, would swell very much the ranks of unemployment, and would spell greater hardships than no compensation at all.

Lastly, I think, perhaps, the most practical solution of the difficulty, in regard to workmen's compensation, would be to include accidents among the benefits of the present Insurance Act. The only important difference this would make would be that employers would pay their premiums to the State instead of to the Insurance Companies.

The latter, who always aver that Workmen's Compensation does not pay them, would no doubt be up in arms at this interference with their widespread philanthropy.

---

